# The role of the endothelial nitric oxide synthase variants in the development of juvenile idiopathic arthritis and its clinical findings

**DOI:** 10.1590/1806-9282.20250835

**Published:** 2026-03-30

**Authors:** Seyda Dogantan, Sevde Hasanoğlu Sayin, Burcu Bozkaya Yücel, Sema Nur Taskin, Khumara Vagif Qizi, Sacide Pehlivan

**Affiliations:** 1 Başakşehir Çam ve Sakura City Hospital, Department of Pediatric Rheumatology – Istanbul, Türkiye.; 2 Istanbul University, Faculty of Medicine, Department of Medical Biology – Istanbul, Türkiye.; 3 Samsun Research and Education Hospital, Department of Pediatric Rheumatology – Samsun, Türkiye.; 4 Eskişehir City Hospital, Department of Pediatric Rheumatology – Eskişehir, Türkiye.

**Keywords:** Juvenile idiopathic arthritis, eNOS, Gene polymorphism, VNTR, PCR, RFLP

## Abstract

**OBJECTIVE:**

Juvenile idiopathic arthritis is the most common chronic arthritis in children and adolescents. Nitric oxide mediates autoimmune responses and plays a role in the etiology of numerous rheumatic diseases. Endothelial nitric oxide synthase regulates nitric oxide levels through catalyzing its production. Here, we evaluated the effect of *eNOS* rs1799983 and variable number tandem repeat variants on disease occurrence and clinical findings in Turkish juvenile idiopathic arthritis patients.

**METHODS:**

One hundred and eight participants, including 58 juvenile idiopathic arthritis patients and 50 healthy controls, were included in the study. *eNOS* rs1799983 and variable number tandem repeat variants were genotyped using polymerase chain reaction and/or restriction fragment length polymorphism. The genotype distribution of the patients was investigated according to their clinical and laboratory results. The results of the analyses were evaluated for statistical significance.

**RESULTS:**

The *eNOS* genotype distributions did not differ between juvenile idiopathic arthritis patients and controls. Within the patient population, those carrying the rs1799983 GT/TT genotypes had higher body mass index, whereas those with the VNTR 4b/4b genotype had lower sedimentation rate.

**CONCLUSION:**

Our study results suggest that the *eNOS* rs1799983 and variable number tandem repeat variants may play a role in juvenile idiopathic arthritis patients’ clinical findings. These data need to be confirmed in different ethnic and larger sample groups.

## INTRODUCTION

Juvenile idiopathic arthritis (JIA) is defined as a noninfectious, autoimmune inflammatory joint disease^1^.

Nitric oxide (NO) is a potent mediator involved in many biological reactions, such as autoimmune reactions and inflammation^2^. NO levels increase in rheumatoid arthritis (RA) patients, and its excessive production is caused by the stimulation of pro-inflammatory cytokines such as interferon alpha (IFN-α), interferon gamma (IFN-γ), tumor necrosis factor alpha (TNF-α), and interleukin-1 beta (IL-1β)^3^. Mammals can produce NO through three distinct isoforms of the NO synthase enzymes, including neuronal synthase (nNOS or NOS1), inducible NOS (iNOS or NOS2), and endothelial NOS (eNOS or NOS3)^4^. *eNOS* (MIM: 163729; GenBank ID: 4846), located on chromosome 7q35-q36, is a possible candidate for controlling the immunological response because it influences NO production^3^. A functional variant in human *eNOS*’s exon 7 is associated with a Glu-Asp alteration at codon 298 (Glu298Asp, also known as G894T or rs1799983). When compared to GG homozygotes, 894T (298Asp) allele carriers have decreased eNOS enzyme activity^5^. A variable number of tandem repeats (VNTR, 27 nt) in intron 4 is another functional variant that contributes to more than 25% of basal plasma NO production^5^. Numerous investigations have demonstrated the correlation between *iNOS* and *eNOS* gene variants and a wide range of diseases, including autoimmune, infectious, and cardiovascular diseases^6,7^.

Considering NO’s role in inflammatory modulation, eNOS variants may contribute to heterogeneity in disease activity and systemic manifestations, even in the absence of direct association with susceptibility. We examined rs1799983 and VNTR (4a/b) to evaluate their potential role in JIA risk and clinical features in the Turkish JIA population. These eNOS variants were chosen due to prior links with altered NO production and inflammation in autoimmune diseases.

## METHODS

### Study population

The study population comprised 58 patients between 1-month and 18-years old, diagnosed with JIA according to ILAR criteria^8^, and 50 age- and sex-matched healthy controls. Patient data were retrospectively collected from two centers: the Department of Pediatric Rheumatology at Başakşehir Çam and Sakura City Hospital (Istanbul) and Samsun University Training and Research Hospital (Samsun), in Turkey. The study was conducted under ethical approval obtained from Samsun Training and Research Hospital (GOKAEK 2024/23/17). While missing prospective power calculations, post-hoc power analysis revealed that our sample size provided 80% power to detect odds ratios ≥2.5 at α=0.05, confirming this pilot study’s adequacy for detecting moderate to large genetic effects.

Individuals with other autoimmune illnesses, cancer, or recent infections were excluded. Every JIA patient had a comprehensive clinical examination and a full history, with particular attention paid to the pattern and distribution of joint involvement, uveitis, and other extra-articular abnormalities and medications. The control group was selected from healthy individuals with no chronic or autoimmune disease history. All patients submitted informed written consent before enrolling in the study, based on the ethical guidelines of the 2008 Declaration of Helsinki.

### Genotyping analysis

DNA was extracted from leukocytes of 5 mL peripheral blood according to the standard protocol. Two *eNOS* gene variants (rs1799983 and intron 4 VNTR) were determined by polymerase chain reaction (PCR) and/or restriction fragment length polymorphism^9^.

For rs1799983, the following primers were used for PCR: 5'-CATGAGGCTCAGCCCCAGAAC-3' (forward) and 5'-AGTCAATCCCTTTGGTGCTCAC-3 (reverse). PCR products were digested overnight at 37°C using MboI (Invitrogen, Carlsbad, CA, USA). Fragments were separated on 3% agarose gel electrophoresis and visualized using ultraviolet light. The 206-bp products had a consistent restriction site, resulting in a 119-bp fragment and an 87-bp fragment.

The VNTR in intron 4 was evaluated with PCR using the following primers: 5'-AGGCCCTATGGTAGTGCCTTT-3' (forward) and 5'-TCTCTTAGTGCTGTGGTCAC-3' (reverse). The amplified products were separated by electrophoresis on a 2% agarose gel and visualized by ethidium bromide staining. The wild-type allele contained five tandem repeats of 27 bp and 420 bp, and the mutant allele contained four tandem repeats of 27 bp and a 393 bp band.

### Statistical analysis

Statistical analyses were performed using Jamovi 2.6.44 and JASP 0.19.3. Continuous variables were assessed for normality (Shapiro-Wilk test) and presented as median [min–max] due to non-normal distribution. Between-group comparisons utilized the Mann-Whitney U test for continuous and the chi-square/Fisher’s exact test for categorical variables.

Hardy-Weinberg equilibrium was verified for both eNOS polymorphisms in controls. Disease risk associations were evaluated using odds ratios with 95%CIs. For genotype-phenotype analyses, VNTR genotypes were grouped as 4a carriers (4a/4a+4a/4b, n=13) versus 4b/4b (n=45), while rs1799983 genotypes were stratified as GG versus T carriers (GT+TT). Effect sizes were calculated using Hodges-Lehmann median differences with bootstrap-derived confidence intervals.

Univariate and multivariate linear regression identified factors associated with ESR and body mass index (BMI) in JIA patients (n=58). Independent variables included demographics, disease characteristics, laboratory parameters, treatment, and eNOS genotypes. Variables with p<0.20 in univariate analysis or clinical relevance entered multivariate models. Model assumptions were verified through residual analysis and multicollinearity assessment (VIF<10). Statistical significance was set at p≤0.05, without multiple testing correction.

## RESULTS

In the present study, no significant differences were observed between groups regarding age, sex, height, weight, and BMI. Median disease duration was 2.0 [1.0–15.0] years, with 58.6% having oligoarticular JIA and 41.4% having enthesitis-related arthritis. Treatment consisted of adalimumab (58.6%) and etanercept (41.4%), with 84.5% showing early treatment response.

The distribution of VNTR genotypes did not differ between groups (4b/4b: 77.6 vs. 76.0%, p=0.999). Similarly, the G894T polymorphism genotype distribution was comparable between groups (GG: 58.6 vs. 46.0%, p=0.290) (Table 1).

**Table 1:** Genetic variants in study groups.

Variables	JIA patients (n=58)	Controls (n=50)	Effect size (95%CI)	p
eNOS VNTR genotype				
4a/4a	1 (1.7)	1 (2.0)	OR 0.86 (0.05–14.11)	0.999
4a/4b	12 (20.7)	11 (22.0)	OR 0.92 (0.37–2.30)	
4b/4b	45 (77.6)	38 (76.0)	OR 1.00 (Reference)	
Dominant model (4a carriers vs. 4b/4b)	13 (22.4)	12 (24.0)	OR 0.91 (0.37–2.24)	0.845
eNOS G894T genotype				
GG	34 (58.6)	23 (46.0)	OR 1.00 (Reference)	0.290
GT	19 (32.8)	24 (48.0)	OR 0.54 (0.24–1.19)	
TT	5 (8.6)	3 (6.0)	OR 1.13 (0.25–5.10)	
Dominant model (T carriers vs. GG)	24 (41.4)	27 (54.0)	OR 0.60 (0.28–1.29)	0.190

Data are presented as n (%). eNOS, endothelial nitric oxide synthase; VNTR, variable number tandem repeat; JIA, juvenile idiopathic arthritis; CI: confidence interval; OR: odds ratio. GG, GT, and TT represent the homozygous G/G (wild-type), heterozygous G/T, and homozygous T/T (variant) genotypes, respectively.

Within the JIA cohort, VNTR 4a carriers demonstrated significantly higher erythrocyte sedimentation rate (ESR) compared to 4b/4b homozygotes (median 15.0 vs. 5.0 mm/h, MD: -10.0, 95%CI -17.0 to -2.0, p=0.039) (Table 2). For the rs1799983 polymorphism, T-allele carriers showed higher BMI compared to GG homozygotes, with a trend toward significance.

**Table 2:** Comparison of clinical parameters by eNOS genotypes in JIA patients (n=58).

Variable	eNOS VNTR		p	eNOS rs1799983		p
	4a carriers (n=13)	4b/4b (n=45)		GG (n=34)	T carriers (n=24)	
BMI (kg/m^2^)						
Median [range]	19.0 [13.0–23.0]	19.0 [14.0–46.0]	0.49	18.0 [13.0–27.0]	20.0 [14.0–46.0]	0.06
MedDiff (95%CI)	0.0 (-2.0, 3.0)			2.0 (0.0, 4.0)		
ESR (mm/h)						
Median [range]	15.0 [4.0–50.0]	5.0 [2.0–77.0]	**0.04**	9.5 [2.0–77.0]	5.0 [2.0–54.0]	0.29
MedDiff (95%CI)	-10.0 (-17.0, -2.0)			-4.5 (-10.0, 1.0)		
CRP (mg/L)						
Median [range]	3.0 [2.0–120.0]	3.0 [1.0–126.0]	0.33	3.0 [1.0–126.0]	3.0 [1.0–110.0]	0.64
MedDiff (95%CI)	0.0 (-1.0, 2.0)			0.0 (-1.0, 1.0)		
WBC (/mm^3^)						
Median [range]	8,880.0 [5,550.0–16,520.0]	8,860.0 [4,810.0–20,200.0]	0.59	9,015.0 [5,550.0–20,200.0]	8,585.0 [4,810.0–17,770.0]	0.33
MedDiff (95%CI)	-20.0 (-2,130.0, 2,090.0)			-430.0 (-2,470.0, 1,610.0)		
Hemoglobin (g/dL)						
Median [range]	12.0 [10.2–15.0]	12.4 [9.4–15.7]	0.56	12.3 [9.4–15.0]	12.3 [9.8–15.7]	0.78
MedDiff (95%CI)	0.4 (-0.3, 1.1)			0.0 (-0.6, 0.6)		
Platelet (/mm^3^)						
Median [range]	3.50×10^5^ [2.06×10^5^–4.66×10^5^]	3.62×10^5^ [1.84×10^5^–9.83×10^5^]	0.90	3.50×10^5^ [2.06×10^5^–9.83×10^5^]	3.635×10^5^ [1.84×10^5^–5.5×10^5^]	0.86
MedDiff (95%CI)	1.20×10^4^ (-4.30×10^4^, 6.70×10^4^)			1.35×10^4^ (-4.00×10^4^,6.70×10^4^)		
Disease duration (years)						
Median [range]	2.0 [1.0–10.0]	2.0 [1.0–15.0]	0.34	2.0 [1.0–10.0]	2.0 [1.0–15.0]	0.39
MedDiff (95%CI)	0.0 (-1.0, 1.0)			0.0 (-1.0, 2.0)		

Bold p-values indicate statistical significance (p≤0.05). Hodges-Lehmann median differences with 95% CI from bootstrap analysis. VNTR 4a carriers include 4a/4a (n=1) and 4a/4b (n=12); rs1799983 T carriers include GT (n=19) and TT (n=5). BMI: body mass index; ESR: erythrocyte sedimentation rate; CRP: C-reactive protein; WBC: white blood cell; CI: confidence interval; eNOS, endothelial nitric oxide synthase; VNTR, variable number tandem repeat.

Univariate analysis of factors affecting ESR revealed significant associations with Juvenile Arthritis Disease Activity Score-27 (JADAS-27) score (p<0.001) and C-reactive protein (p<0.001), while eNOS VNTR genotype (p=0.717), age (p=0.202), sex (p=0.092), and other variables showed no significance. In the multivariate model, only JADAS-27 (β=1.00, p<0.001) and C-reactive protein (β=0.33, p<0.001) remained as independent predictors (R^2^=0.845, p<0.001) (Table 3). Regression analysis for BMI demonstrated significant univariate associations with age (p<0.001), sex (p=0.012), disease subtype (p<0.001), treatment modality (p=0.029), and eNOS G894T genotype (p=0.001). The multivariate model identified age (β=0.30, p<0.001), disease subtype (β=-3.76, p=0.013), and eNOS G894T genotype (β=3.08, p=0.026) as independent predictors (R^2^=0.432, p<0.001) (Table 3).

**Table 3:** Univariate and multivariate linear regression analysis of factors associated with erythrocyte sedimentation rate in children with juvenile idiopathic arthritis.

Variables	ESR model		BMI model	
	β (95%CI)	p	β (95%CI)	p
Age (years)	-0.24 (-1.17, 0.70)	0.289	0.30 (0.19, 1.56)	**<0.001**
Sex (male vs. female)	-2.31 (-5.59, 0.97)	0.168	1.86 (-1.22, 4.90)	0.117
Disease subtype (ERA vs. oligo)			-3.76 (-5.91, -1.61)	**0.013**
JADAS-27 score	1.00 (0.19, 1.82)	**<0.001**	–	–
C-reactive protein (mg/L)	0.33 (0.05, 0.62)	**<0.001**	–	–
Treatment (etanercept vs. adalimumab)	–	–	1.55 (-1.38, 4.48)	0.332
eNOS VNTR (4a carriers vs. 4b/4b)	2.35 (-2.30, 7.01)	0.246	–	–
eNOS G894T (T carriers vs. GG)	–	–	3.08 (1.36, 4.86)	**0.026**
Model R^2^	0.845		0.432	
Adjusted R^2^	0.826		0.365	
F-statistic	F(5, 52)=46.2	**<0.001**	F(5, 52)=6.48	**<0.001**

Bold p-values indicate significance (p≤0.05). Only variables with p<0.20 in univariate analysis or clinical relevance were included in multivariate models. ESR: erythrocyte sedimentation rate; BMI: body mass index; CI: confidence interval; ERA: enthesitis-related arthritis; JADAS-27: Juvenile Arthritis Disease Activity Score-27; eNOS: endothelial nitric oxide synthase; VNTR: variable number tandem repeat.

## DISCUSSION

Although the exact etiology underlying JIA remains unclear, genetic predisposition, immune dysregulation, and environmental triggers have been implicated, including increased oxidative stress (OS) in children with JIA^10^. NO is a key link between inflammation and OS, acting as both a pro-inflammatory mediator and vascular regulator. Consistently, RA patients have higher blood and synovial fluid NO concentrations than healthy individuals^11^. While NO generation in adult joint tissue is well linked to arthritis, pediatric studies remain limited^12,13^.

Two *eNOS* variants, rs1799983 (exon 7) and intron 4 VNTR (4a/b), are known to influence enzyme activity and NO levels^3^. Among the rs1799983 variants, T-allele carriers have reduced eNOS activity^3^. Regarding the intron 4 VNTR, the 4a allele lowers NO levels^3^ and has been linked to RA^14^. Bunjevacki et al. suggested that *eNOS* variants are associated with disease activity rather than susceptibility, and minor alleles are indicators of a better clinical course^3^. Similarly, studies by Gonzalez-Gay et al.^15^ (on T-786C and rs1799983 variants) and AlFadhli^16^ (on VNTR genotype and allele distribution) found genotype or allele frequencies were comparable between RA patients and controls. Pehlivan et al. associated rs1799983 TT genotype, but not VNTR, with RA^17^. Based on this information, we evaluated the effect of *eNOS* variants on the disease and clinical findings in Turkish JIA patients. To the best of our knowledge, this is the first study evaluating such associations in Turkey.

Our results showed similar genotype distributions between all participants, unlike a previous report in a Greek population associating VNTR polymorphism with JIA susceptibility^18^. However, genetic regulation may differ between JIA and RA; for example, T-786C increased RA risk in a Chinese cohort^19^, while other studies linked *eNOS* variants to disease activity rather than susceptibility^20^. Similarly, a previous meta-analysis failed to indicate any association between RA and eNOS 4b/a^21^. Nevertheless, our study focuses on Turkish pediatric JIA patients whose ethnic and disease profiles differ from adult RA, which may explain global discrepancies.

To explore clinical relevance, we analyzed *eNOS* variants using univariate and multivariate approaches. The higher BMI trend in rs1799983 GT/TT carriers reached significance in multivariate analysis and remained after adjusting for covariates (β=3.08, 95%CI 1.36–4.86, p=0.026). This suggests an effect independent of genotype, though unmeasured factors such as corticosteroid use or physical activity may contribute to this relationship. Mechanistically, the interplay between high BMI and rs1799983 T-allele carriers may be explained through impaired the PI3K–Akt–eNOS pathway, leading to diminished NO generation and reduced insulin-mediated vasodilation^22^. This results in limited blood flow and capillary recruitment, thereby decreasing glucose and insulin delivery to peripheral tissues. Conversely, endothelial dysfunction characterized by reduced NO bioavailability can itself promote insulin resistance, establishing a vicious cycle that exacerbates metabolic abnormalities. This observation underscores the importance of monitoring metabolic parameters in genetically at-risk JIA patients.

The patients with the VNTR 4b/4b genotype exhibited a lower ESR than patients with 4a carriers. Interestingly, this association did not persist in multivariate regression after adjusting for disease activity markers. The multivariate model identified CRP (β=0.33, p<0.001) and JADAS-27 score (β=1.00, p<0.001) as the primary determinants of ESR, with the VNTR genotype effect becoming non-significant (p=0.246), suggesting the VNTR–ESR association likely reflects disease activity differences rather than the genotype. The strong correlation between ESR and disease activity emphasizes the importance of clinical context when interpreting genotype-phenotype associations in inflammatory conditions. These findings are in alignment with profiles observed in RA, where reduced eNOS activity is associated with elevated ESR, which in turn correlates with cardiovascular morbidity^23^. Genetic variation in *eNOS* may influence inflammatory biomarker levels and, potentially, cardiovascular risk in JIA patients, highlighting the importance of cardiovascular monitoring in this population.

Several limitations should be acknowledged here. The cross-sectional design limits causal inference, and incomplete corticosteroid histories reduce our ability to assess treatment effects. Lifestyle factors such as diet and activity, which may influence BMI and inflammation, were not captured. Finally, the absence of plasma NO measurements leaves mechanistic genotype–phenotype links to be clarified in future work.

This study’s strengths include a well-characterized pediatric cohort, careful genotyping, and structured clinical evaluation. If confirmed in larger cohorts, these findings may help identify JIA subgroups with distinct inflammatory or metabolic profiles, supporting personalized management. Future work should track medications, physical activity, and NO metabolites and assess the impact of eNOS variants on NO production and endothelial function to strengthen biological plausibility.

## CONCLUSION

In conclusion, although the rs1799983 and VNTR variants were not associated with disease susceptibility in Turkish children with JIA, certain genotypes were related to BMI and ESR. These findings suggest a potential role for *eNOS* variants in modulating disease phenotype and merit further validation in larger, multi-ethnic populations.

## Data Availability

The datasets generated and/or analyzed during the current study are available from the corresponding author upon reasonable request.
